# Prevalence and associated factors influencing stunting in children aged 2–5 years in the Gaza Strip-Palestine: a cross-sectional study

**DOI:** 10.1186/s12887-017-0957-y

**Published:** 2017-12-21

**Authors:** Rima Rafiq El Kishawi, Kah Leng Soo, Yehia Awad Abed, Wan Abdul Manan Wan Muda

**Affiliations:** 1 School of Public Health, Al Quds University, Gaza City, Gaza Strip Palestine; 2 Program of Nutrition, School of Health Sciences, Health Campus, Universiti Sains Malaysia, 16150 Kubang Kerian, Kelantan Malaysia

**Keywords:** Stunting, Prevalence, Associated factors, Gaza strip

## Abstract

**Background:**

Stunting continues to be a major public health problem in developing countries. It is one of the most important risk factors for morbidity and mortality during childhood. In Palestine, it is another health problem, which adds to the catastrophic issues in the region. This study aimed to determine the prevalence of stunting and its associated factors among preschool children in the Gaza Strip.

**Methods:**

A cross-sectional study design was conducted in the Gaza Strip. A total of 357 children aged 2–5 years and their mothers aged 18–50 years were recruited. A multistage cluster sampling was used in the selection of the study participants from three geographical areas in the Gaza Strip: Jabalia refugee camp, El Remal urban area, and Al Qarara rural area. A structured questionnaire was used for face- to -face interviews with the respective child’s mother to collect sociodemographic information and feeding practice. Anthropometric measurements for children were taken to classify height-for-age (HAZ), while maternal height was measured as well. Descriptive and binary logistic regression analyses were applied to determine the prevalence and associated factors with stunting.

**Results:**

The total prevalence of stunting in this study was 19.6%, with the highest prevalence being (22.6%) in Jabalia refugee camp. It turns out that shorter mothers had increased the odds of stunting in preschool children in the Gaza Strip. Children born to mothers whose height was 1.55–1.60 m or <1.55 m were more likely to be stunted (*p* = 0. 008), or (*p* < 0.001), respectively, than children born to mothers whose height was >1.60 m. Moreover, parental consanguinity increased the risk of stunted children (*p* = 0. 015).

**Conclusions:**

This study showed the prevalence of stunting was of alarming magnitude in the Gaza Strip. Our results also demonstrated that parental consanguinity and short maternal stature were associated with stunting. Culturally appropriate interventions and appropriate strategies should be implemented to discourage these types of marriages. Policy makers must also raise awareness of the importance of the prevention and control of nutritional problems to combat stunting among children in the Gaza Strip.

**Electronic supplementary material:**

The online version of this article (10.1186/s12887-017-0957-y) contains supplementary material, which is available to authorized users.

## Background

Malnutrition is a major health problem in most developing countries. Despite the improvement in health status of children aged less than 5 years in developing countries, undernutrition remains a significant public health problem [1]. Worldwide, it was estimated that one in every three preschool children is malnourished. In 2012, an estimation of 162 million children under-5 year olds were stunted, 99 million were underweight and 51 million were wasted, and 17 million were severely wasted [2]. Inadequate nutrition in the first 1000 days of a child’s life can lead to stunted growth, which is irreversible [3]. The global prevalence in stunting and numbers of children affected is decreasing. Between 2000 and 2012 stunting prevalence declined from 33.0% to 25.0% and the numbers declined from 197 million to 162 million. In 2012, about half of all stunted children lived in Asia and over one-third in Africa [2]. Stunting (low height for age) refers to a failure to reach linear growth potential; those children falling below two standard deviations of the reference population are at high risk [4]. The main consequences of poor growth in childhood can be classified in terms of mortality, morbidity, mental and intellectual development. Important adverse outcomes in adult life, such as body size, work performance, reproductive performance, and risk of acquiring chronic diseases, are also affected by childhood growth [5]. A baseline assessment of dietary intake and nutritional status in September 2002 revealed that prevalence of stunting was 17.5% among Palestinian children aged between 6 and 59 months in the West Bank and the Gaza Strip [6]. The results of the nationwide Palestinian Family Health Surveys indicated that, in 2006, the prevalence of stunting in children younger than 5 years was 8.5% in the West Bank and 15.3% in the Gaza Strip [7]. Chronic malnourishment was noticeably observed among refugee children and was worse among those in the Gaza Strip (13.2%) compared with those in the West Bank (10.6%) [8]. That could be attributed to deterioration economic status in the Gaza Strip. Numerous possible causes of malnutrition were categorized into three levels: namely, the basic level; the underlying or intermediate level; and, the direct level as classified within the United Nations Children’s Fund (UNICEF) framework [9]. The influence of parental characteristics such as consanguinity has not been explored fully within the UNICEF framework. Consanguinity is an important concern affecting the health status of offspring and children. Consanguinity is associated with high prevalence of recessive features and diseases, some of which may negatively affect weight and height of children [10]. A previous study in Palestine showed a high prevalence of consanguineous marriage [11]. In the Gaza Strip, few studies have been conducted on stunting among preschool children. Thus, this study aimed to determine the prevalence of stunting in children aged 2–5 years in the Gaza Strip and its associated factors. We hypothesized that child stunting would be associated with consanguinity when taking into consideration maternal and sociodemographic factors.

## Methods

This study was conducted in three areas in the Gaza Strip, namely, Jabalia refugee camp, El Remal urban area, and Al Qarara rural area. A cross-sectional design was carried out to recruit a total of 357 children aged 2-5 years and their mothers aged 18–50 years. The study was conducted from April to October 2012.

The single proportion formula was used to calculate the sample size; a sample of 334 participants was selected with a confidence level of 95.0%. Accounting for an attrition rate 20.0%, the total number of participants was calculated as follows: 334 + (0.20 × 334) = 400. Therefore, 400 participants were recruited for the study from the three different geographical areas in the Gaza Strip. Inclusion criteria included being a mother aged 18–50 years with a child aged 2-5 years residing in one of the three different sociodemographic areas in the Gaza Strip, namely, Jabalia refugee camp, El Remal urban area, and Al Qarara rural area. Children were excluded if they suffered from psychomotor retardation, hormonal disorders, chronic debilitating diseases, congenital heart diseases, and acute severe illnesses. In households with more than one child aged 2–5 years, the youngest child was selected.

### Sampling method

Multistage cluster sampling was used to recruit the study participants. At the first stage, the numbers of areas were selected randomly from the entire clusters, namely, urban area, refugee camp, then from the rural area. At the second stage, households were systematically selected within each cluster in the urban, the refugee camp, and the rural area respectively. The number of households chosen for each cluster was weighted in proportion to the total population of children aged 2–5 years in each area. The percentage of preschool children was estimated at 19.2% of the total population. A total of 220, 140 and 40 households were selected from Jabalia refugee camp, El Remal urban area, and Al Qarara rural area, respectively. The number of households successfully recruited was 357, yielding a household response rate of 89.2%. Of the 43 non-respondents, 12 mothers refused to participate in the study, 15 households excluded children ages 2–5 years, and 16 children refused anthropometric measurements.

A mother of a child aged 2–5 years was selected for an interview from each household; each interviews took approximately 30 min. A structured questionnaire was used to collect sociodemographic information and feeding practices of children (Additional file 1: Appendix A). Anthropometric measurements were taken by two trained research assistants following standard recommended procedures of the World Health Organization (Additional file 1: Appendix B). Children were weighed with a SECA portable calibrated electronic scale (precision of 100 g). The researcher calibrated the scale before each measurement session. Accuracy was checked by comparing the scale reading with a known weight. The child was weighed barefoot, wearing only underwear. The measurements were taken twice and the average was calculated. The heights of the children were measured using non-stretchable constant tapeline, with 0.1 cm precision. The child was instructed to remove his/her shoes. The height was then measured while standing against a wall with feet flat on the base, the heels, buttocks, shoulders, and back of the head touching the wall, and the head positioned looking straight ahead. The mean of two measurements was calculated.

The children’s ages were calculated in months and based on their birth certificates. To assess children’s nutritional status, anthropometric data were transformed into Z-scores using the program WHO ANTHRO (version 3.2.2, January 2011) [12]. Finally, consistency across indicators was checked and tested before the results were entered into the computerized system. The researcher used WHO classification [13] to assess the nutritional status of children. The following definitions were used in this study:

- Stunting (low height for age) is defined as a Z-score < −2 SD of the reference population. It refers to a chronic nutritional disorder.

Heights for mothers were measured in meters using a portable body meter with 0.1 cm precision. The respondent stood without shoes against a wall, with feet flat on the base, the heels, buttocks, shoulders, and back of the head touching the wall, and the head positioned looking straight ahead. The mean of two measurements was calculated.

### Data analysis

The Statistical Package for Social Science (SPSS), version 22 was used to analyze the study data. The descriptive data were expressed as mean ± standard deviation (SD). The Chi-square test was conducted to determine the differences between the proportions of stunting in the three geographical locations.

Determinants of stunting were examined using binary logistic regression. The dependent variable was stunting (Z-score less than 2SD). While the independent determinants were:

- Child’s age, and sex.
- Mother’s and father’s education were categorized into low level of education (illiterate, primary school, and preparatory school), moderate level of education (secondary school), and high education level (graduate or postgraduate university).
- Mother’s employment was identified as working or housewife, and father’s job was identified as working or not working.
- Household’s monthly income.
- Household’s size.
- Mother’s height was measured in meters and categorized as <1.55 m, 1.55–1.60 m, or >1.60 m.

-Consanguinity was categorized as follows:

Yes: there is blood relationship (First cousin: it means that the closest ancestor that two people have in common is a grandparent, and first cousin once removed: It means that the person is married to the children of his/her cousins).

No: There is no blood relationship.

- Mother’s age at the birth of her child categorized as: (<20 years of age, 20–30 years, or >30 years).
- Child’s birth order (the child’s birth order is the position of child birth order regarding his/her siblings in the household).
- Breastfeeding practices.

In binary logistic regression model, the differences were considered to be statistically significant when the *p*-value obtained was <0.05.

### Ethical issues

Ethical approval is required before starting data collection including pilot study. Ethical approval for the study was obtained from University Sains Malaysia Ethical Committee and the Helsinki Committee of the Ministry of Health in the Gaza Strip. Informed written consent was obtained from the participants prior to their participation. The informed consent stated the purpose of the study, study confidentiality, and the voluntary right of participation in the study, as well as provided the guarantee that no participant suffered any harm as a result of his/her participation in the study.

### Pilot study

Prior to conducting this study, pilot testing was performed on 30 mothers from the three geographical areas in the Gaza Strip to assess the validity of the instrument and the value of the questions to elicit the right information, and to determine the ability of the respondents to complete the questionnaire within the time frame (Additional file 1: Appendix A). In addition, participant written consent forms were also tested for comprehension. The pilot study participants were not included in the study.

## Results

Table 1 presents the background characteristics of participants. The majority of children lived in a refugee camp (60.8%), 28.0% lived in an urban area, while the smallest percent lived in a rural area (11.2%). More than half of the children were boys and the rest were girls. The highest proportion of children were between 24 and 35 months, and the mean of child’s birth order was ≈ 4.0 **±** 2.34 month. Monthly income was categorized into three categories the first one was more than 1400 shekel (359US $), then 1000–1400 (256-359US$), and the last one less than 1400 shekel (359 US $). Almost all children were breastfed and 24.4% received only breast milk up to 6 months. Among mothers, the highest percent (41.1%) were >1.60 m tall, and 35.9% were between 1.55 and 1.60 m tall, while the 23.0% were <1.55 m tall. Regarding consanguinity between parents, results showed that 38.7% of parents shared blood relatives. Table 2 presents the anthropometric data of preschool children taken during the survey. Mean child’s body weight was 14.20 ± 2.42 kg, and mean child’s height was 94.14 ± 7.94 cm. Based on results in Table 3, the proportion of stunting among the children was 19.6%, and the highest proportion of stunting was in Jabalia refugee camp (22.6%), in El Remal urban area, stunting was 17.0%; and, in Al Qarara rural area, stunting was 10.0%. There was no significant association between the three geographical areas (*p*-value >0.05). There were variables influencing the prevalence of stunting in the Gaza Strip. Results in Table 4 showed the associated determinants of stunting in preschool children. Mother’s height had a significant influence on the odds of stunting. Children born to mothers whose height was 1.55–1.60 m or <1.55 m were more likely to be stunted (OR_adj_, 2.66, 95% CL, 1.29, 5.46; *p* = 0. 008), or (OR_adj_, 6.38, 95% CL, 3.07, 13.26; *p* < 0.001), respectively, than children born to mothers whose height was >1.60 m. Children whose parents had blood relatives were at a higher risk for stunting (OR_adj_, 1.98, 95% CL, 1.14, 3.44; *p* = 0. 015) compared to children whose parents were not blood relations. Other variables found not to be significantly associated with the stunting were: geographical location, educational levels of mothers and fathers, child’s sex, age, monthly income, breastfeeding, and age of mother at time of birth.

**Table 1:** General characteristics of participants (*n* = 357)

Variables	Frequency (*n*)	Percent (%)
Geographical location		
Urban area	100	28.0
Refugee camp	217	60.8
Rural area	40	11.2
Child’s sex		
Boy	188	52.7
Girl	169	47.3
Child’s age (month) Mean 39.58 ± 10.74		
24–35	153	42.9
36–47	111	31.1
48–60	93	26.0
Child’s birth order Mean 3.99 ± 2.34		
Mother’s age at child’s birth (year) Mean 30.80 ± 6.39		
> 30.0	111	31.1
20–30	203	56.9
< 20.0	43	12.0
Mother’s educational level		
Illiterate & Elementary	20	5.6
Preparatory	118	33.1
Secondary	140	39.2
University Graduate	79	22.1
Mother’s job		
Employed mother	18	5.0
Housewife	339	95.0
Father’s educational level		
Illiterate & Elementary	29	8.1
Preparatory	100	28.0
Secondary	95	26.6
University Graduate	123	34.5
Post graduate	10	2.8
Father’s job		
Working	275	77.0
Not working	82	23.0
Household size Mean 6.50 ± 1.99		
Monthly income (Shekel)*		
> 1400	109	30.5
1000–1400	107	30.0
< 1400	141	39.5
Breastfeeding		
Yes	349	97.7
No	8	2.3
Exclusive Breastfeeding*		
Yes	87	24.4
No	270	75.6
Mother’s height (m) Mean 1.59 ± 0.06		
> 1.60	147	41.1
1.55–1.60	128	35.9
< 1.55	82	23.0
Consanguinity between parents		
Yes	138	38.7
No	219	61.3

*1$US = 3.9Shekel

*Exclusive breastfeeding in the first six month

**Table 2:** Child Malnutrition (*n* = 357)

Variables	Frequency (*n* = 357)	Percent (%)	Mean (SD)
Child’s body weight/kg			14.20(2.42)
Child’s body height/cm			94.14(7.94)
Height for Age (HAZ)			
Normal (−1.0-to 2.0)	170	47.6	
Mild Stunting (−2.0-to < −1.0)	117	32.8	
Moderate Stunting (−3.0 ≤ to <−2.0)	70	19.6

**Table 3:** Child malnutrition in the three different geographical areas

Variables	Urban	Rural	Refugee camp	*P*-value
	(*n* = 100)(%)	(*n* = 40)(%)	(*n* = 217) (%)	
Stunting	17(17.0)	4(10.0)	49(22.6)	0.136
Normal	83(83.0)	36(90.0)	168(77.4)

**Table 4:** Associated factors of stunting in children in the Gaza Strip (*n* = 357)

Variables	Simple logistic regression			Binary logistic regression^a^		
	B	Crude OR (95% CI)	*P*-value	Exp(B)	Adjusted OR (95% CI)	*P*-value
Geographical location_1_	−0.61	0.54(0.17,1.72)	0.300	–	–	–
Geographical location_2_	0.35	1.42(0.77,2.62)	0.257	–	–	–
Child age	−0.01	0.98(0.96,1.01)	0.224	–	–	–
Child’s sex	−0.37	0.69(0.40,1.17)	0.172	–	–	–
Mother’s age at child birth_1_	0.71	1.18(0.65,2.17)	0.575	–		
Mother’s age at child birth_2_	0.51	1.66(0.71,3.87)	0.237	–		
Mother’s education_1_	0.79	2.21(1.02,4.76)	0.042	–		
Mother’s education_2_	0.47	1.60(0.72,3.52)	0.242	–		
Family members	0.05	1.05(0.93, 1.18)	0.412	–	–	–
Father’s education_1_	0.34	1.41(0.76,2.65)	0.278	–	–	–
Father’s education_2_	−0.41	0.66(0.34,1.26)	0.210	–	–	–
Household income_1_	0.24	1.27(0.57,2.81)	0.555	–	–	–
Household income_2_	0.70	2.01(0.93,4.35)	0.075	–		
Breastfeeding	0.92	2.52(0.59,10.83)	0.212	–	–	–
Exclusive breastfeeding	0.20	1.22(0.65,2.30)	0.523	–	–	–
Mother’s height_1_	0.96	2.62(1.28,5.36)	0.008	0.97	2.66(1.29,5.46)	0.008
Mother’s height_2_	1.83	6.26(3.04,12.91)	<0.001	1.85	6.38(3.07,13.26)	<0.001
Consanguinity	0.65	1.92(1.13,3.25)	0.015	0.68	1.98(1.14,3.44)	0.015

^a^Forward LR binary Logistic regression model was applied

Model assumption are fulfilled

There were no interactions amongst independent variables. No multicolinearity detected

Hosmer and Lemeshow test (*p* = 0.678)

Classification table (overall correctly classified percentage = 80.4%), Area under the curve 77.6%

Address: Urban is reference. Address_0_, is rural to urban. Address_1_is refugee to urban

Educational High level is the reference. Educational level _1_ is Medium level. Educational level _2_ is Low level

Income >1400Shekel is the reference group, US $ = 3.90 Shekel

Mother’s height > 1.60 m is the reference, 1.55–1.60 m is mother height 1, and <1.55 m is mother’s height 2

## Discussion

Anthropometry is extremely useful in assessing the nutritional status of individuals and populations [14]**.** Anthropometric data can evaluate the general health status, diet, growth, and development of a child over time [15]. Based on our results, the prevalence of stunting among preschool children was 19.6%. Our finding is higher than the result of a previous study that reported a prevalence of stunting of 15.0% in the Gaza Strip, in 2013 [16]. The results of this study indicated that continuing deterioration of the nutritional status among preschool children in the Gaza Strip. In Arab countries, the prevalence of stunting among children younger than 5 years old ranged from 8.0% in Qatar to 53.0% in Yemen [17]. In the present study, it was noticed that the highest prevalence of stunting was in the refugee camp, and contrary to our expectations, we found the prevalence of stunting in the urban area was higher than in the rural area. This result might be explained by poor overall economic conditions, as poverty rate in 2010 in the Gaza Strip was 61.0% [18]. The worst economic conditions in the Gaza Strip have negatively affected the population, particularly children. Refugees and non-refugees in the Gaza Strip were severely affected by the deterioration of socioeconomic conditions [7, 19], in turn, faced heightened food insecurity levels, which exceeded 44.0% of households in 2011 and increased to an alarming 57.0% in 2012 [19]. In our study, food insecurity wasn’t included as an associated factor of stunted children, but numerous studies have reported that food insecurity affects health and well-being throughout the life cycle; in fact, it has been associated with children’s dietary intake and weight status [20, 21]. Household food insecurity may be related to protein energy malnutrition, which was evident in cases of stunting [22, 23]. In the Gaza Strip, the population growth and structure affect the economic development and public services which are already greatly deteriorated. Furthermore, the demand for healthcare services and education will be increased, at the same time. Though socioeconomic conditions have an effect on children’s nutritional status, genetic factors must also be considered [24]. Many studies have rarely included genetic components [25]. In this study, two genetic factors (mother’s height and parental consanguinity) were determined to be important factors influencing stunting of children in the Gaza Strip. The results of the present study revealed that parental consanguinity was positively associated with stunting in children. Parental consanguinity increased the risk of stunting in children. Children born to consanguineous parents are at a high risk of autosomal recessive diseases, and multifactorial disorders [26]. All consequences of consanguinity predispose a child to poor growth and may increase stunting [27, 28]. A previous study conducted in Egypt supported the results of the present study, in which consanguineous marriages influenced stunted children in Egypt [24]. Parental consanguinity was associated with the malnourished child. Consanguinity is associated with increased the risk of congenital anomalies and infant mortality in Pakistan, the relative risk of infant mortality varying between 1.4 and 1.8 for consanguineous compared to non consanguineous marriages [29, 30]. On the other hand, the results of this study showed a decline in the prevalence of stunting as the mother’s height increased. Our results highlight a novel result a**s** no previous studies in the Gaza Strip reported the inverse association between mother’s short stature and stunted children. Few countries have assessed the influencing maternal stature and childhood stunting [31]. These findings are consistent with another study conducted in Egypt that reported mothers’ short stature were more likely to have stunted children [24]. In Mexico, results of the national nutrition survey showed that short stature of mothers was significantly associated with stunted children [32]. The early life factors including mother’s poor health and nutrition stores before, during and after pregnancy are associated with increased child’s growth failure. The long-term impacts of mother’s poor health status, and the inadequate supply of nutrients to her fetus can lead to intrauterine poor growth and low birth weight, which can affect child’s health and survival [32].

### Limitations of the study

Due to limited funds, more related variables were not able to be examined as predictors of stunting. Another limitationis that this cross- sectional study describes only the association of stunting and not the causal relationship. Thus, in-depth case-control studies should be conducted in the future to address the risk factors of stunting in the Gaza Strip.

## Conclusion

Our results provide evidence that consanguineous marriage and maternal height were associated factors for childhood stunting in the Gaza Strip. The results of this study showed an increase in stunting proportion as mother’s height decreased. Moreover, parental consanguinity was associated significantly with increasing rate of stunting. This suggests the presence of an intergenerational transmission from mother’s own nutrition, disease, and socioeconomic circumstances during her childhood to her offspring’s health and mortality in their infancy and childhood.

### Recommendation

More studies are needed to explore the influence of genetic characteristics and environmental factors on childhood nutrition status in the Gaza Strip. In addition, health workers should apply educational programs before marriage among couples to raise awareness about the risk of consanguinity marriage on childhood health status.

## Additional file

Additional file 1: Appendix A. Questionnaire. **Appendix B.** Anthropometric measurements for the mother and the child on the interview's day. (DOCX 19 kb)

## Abbreviations

HAZ: Height-for-age
SD: Standard deviation
SPSS: Statistical Package for Social Science
UNCIF: United Nations Children’s Fund
WHO: World Health organization
