# Supplementary material for: Prevalence and associated factors influencing stunting in children aged 2–5 years in the Gaza Strip-Palestine: a cross-sectional study
Source: BMC Pediatr. 2017 Dec 21;17:210. doi: 10.1186/s12887-017-0957-y (PMC5740756; doi:10.1186/s12887-017-0957-y)
Supplement: Additional file 1: Appendix A. — Questionnaire. Appendix B. Anthropometric measurements for the mother and the child on the interview's day. (DOCX 19 kb) [file 12887_2017_957_MOESM1_ESM.docx]

**Additional file 1.**

**Appendix A. Questionnaire**

|  |
| --- |
| -Serial Number --- -Interview Date:--/--/-- |
| -Telephone / Mobile No… |
| **Socio-demographic** |
| 1-Residency place 1- El Remal area 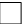 2-Al Qarara 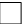 3-Jabalia camp |
| 2-Child’s Sex: 1-Male 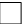 2-Female 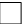 |
| 3-Child’s age in months 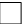 Birth Date: --/--/-- |
| 4-No. of family members who lives in the household 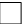 |
| 5-Child’s order/rank among live siblings 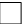 |
| 6-Your age when you had your baby? (in years) 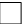 |
| 7-What is your educational level  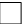 Illiterate 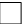 primary 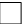 preparatory  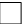 Secondary 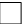 graduated 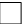 postgraduate |
| 8-Are you working?  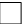Yes 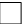 No |
| 8.1 If yes where, specify_______ |
| 9-What is your husband’s educational level  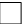 Illiterate 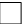 primary 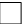 preparatory  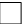 Secondary 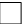 graduated 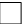 postgraduate |
| 10-Is your husband working?  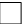Yes 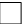 No |
| 10.1 If yes where, specify_______ |
| 11-Family income per month in shekel 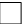  12-Consanguinity (relationship between parents)  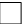Yes 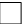 No |

| ***Mother's feeding practice*** |
| --- |
| 13-Did you breast-feed your child? |
| 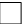Yes 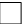No |
| - If yes: |
| 13.1 How long did you breast-feed your child…. |
| 14-Did you exclusively breast-feed your child for the first 6 months? |
| 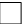 Yes 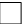No |
| 2.3At 14.1 If No, At what age introducing semi solids or solid-foods into your child's diet? 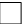 |

**Anthropometric measurements for mother and the child on the interviewer’s day (Appendix B)**

**S/N….**

**Anthropometric measurements for mother on the interview's day**

-Mother’s body height……m

**Anthropometric measurements for child on the interview's day**

-Child’s body weight………kg

-Child’s body height……….cm

**Thank you for your cooperation**
